# Left−Right Asymmetry Defect in the Hippocampal Circuitry Impairs Spatial Learning and Working Memory in *iv* Mice

**DOI:** 10.1371/journal.pone.0015468

**Published:** 2010-11-17

**Authors:** Kazuhiro Goto, Ryo Kurashima, Hayato Gokan, Naomi Inoue, Isao Ito, Shigeru Watanabe

**Affiliations:** 1 Japan Society for the Promotion of Science, Tokyo, Japan; 2 Faculty of Letters, Department of Psychology, Keio University, Tokyo, Japan; 3 Faculty of Sciences, Department of Biology, Kyushu University, Fukuoka, Japan; Université Pierre et Marie Curie, France

## Abstract

Although left-right (L−R) asymmetry is a fundamental feature of higher-order brain function, little is known about how asymmetry defects of the brain affect animal behavior. Previously, we identified structural and functional asymmetries in the circuitry of the mouse hippocampus resulting from the asymmetrical distribution of NMDA receptor GluR ε2 (NR2B) subunits. We further examined the ε2 asymmetry in the *inversus viscerum* (*iv*) mouse, which has randomized laterality of internal organs, and found that the *iv* mouse hippocampus exhibits right isomerism (bilateral right-sidedness) in the synaptic distribution of theε2 subunit, irrespective of the laterality of visceral organs. To investigate the effects of hippocampal laterality defects on higher-order brain functions, we examined the capacity of reference and working memories of *iv* mice using a dry maze and a delayed nonmatching-to-position (DNMTP) task, respectively. The *iv* mice improved dry maze performance more slowly than control mice during acquisition, whereas the asymptotic level of performance was similar between the two groups. In the DNMTP task, the *iv* mice showed poorer accuracy than control mice as the retention interval became longer. These results suggest that the L−R asymmetry of hippocampal circuitry is critical for the acquisition of reference memory and the retention of working memory.

## Introduction

The molecular basis of left-right (L−R) asymmetries in brain structure and function is one of the central issues to be elucidated in neuroscience. L−R asymmetry (laterality) of the brain, once believed to be a human characteristic, has now been found to be widespread among vertebrates [1]–[4]. Conventional laterality research has mainly dealt with asymmetries in higher-order functions and in gross anatomical structures of the brain. This is in part a consequence of the lack of an index for investigating brain asymmetry at the molecular level with experiments in vitro.

**Figure 1:**
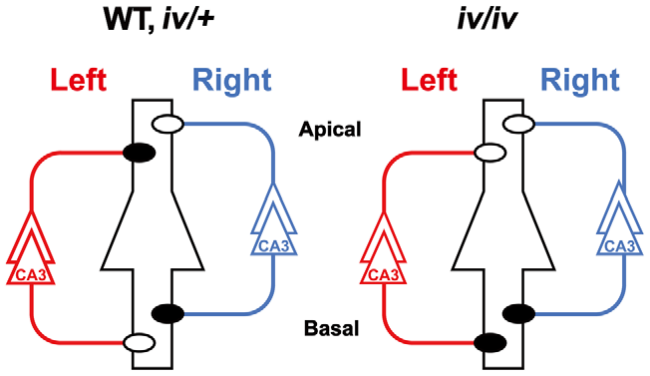
Hippocampal asymmetry and right isomerism of the *iv/iv* mouse hippocampus. Left and right CA3 pyramidal neurons and their axons are colored red and blue, respectively. A postsynaptic CA1 pyramidal neuron is at the center, colored black, and it represents postsynaptic neurons in both left and right hemispheres. Closed and open circles represent ε2-dominant andε2-nondominant synapses, respectively. Apical, apical dendrites; Basal, basal dendrites.

We have previously shown that the distribution of *N*-methyl-D-aspartate receptor (NMDAR) ε2 subunits in the mouse hippocampus is asymmetrical, both between synapses formed on the apical and basal dendrites of individual neurons and between synapses formed by inputs from the left and right pyramidal neurons (Figure 1, WT)[5], [6]. These asymmetrical allocations of ε2 subunits affect the properties of NMDARs in hippocampal synapses and generate two populations of synapses with complementary properties. One population consists of ‘ε2-dominant’ synapses, in which the NMDAR-mediated excitatory postsynaptic currents (NMDA EPSCs) show high sensitivity to Ro 25-6981, an ε2 subunit selective antagonist [7]–[9]; in this population, synaptic plasticity develops rather early. The other population consists of ‘ε2-nondominant’ synapses, in which the NMDA EPSCs are less sensitive to Ro 25-6981; in this population, synaptic plasticity develops slowly. These two populations of synapses are located asymmetrically in the hippocampal circuitry. We hypothesized that the synaptic distribution of ε2 subunits and the properties of NMDAR-mediated synaptic functions might be sensitive and quantitative indices for detecting abnormalities in the L−R asymmetry of the brain.

To investigate the mechanisms for generating hippocampal asymmetry, we examined ε2 asymmetry in the *iv* mouse [10]. *Iv* is a spontaneous mouse mutant that has a mutation in the gene encoding the motor protein, *Left-right dynein (Lrd)* [11]. In 7.5 day postcoitum embryos, the leftward nodal flow generated by the rotation of cilia initiates the L−R axis determination process [12], [13]. In *iv* homozygous (*iv*/*iv*) embryos, however, the nodal cilia are immotile because of the mutation in *Lrd*, and thus fail to produce constant leftward flow, resulting in randomized laterality of visceral organs [14]–[16]. Fifty percent of *iv/iv* mice exhibit reversed asymmetry (*situs inversus*), whereas the rest are normal (*situs solitus*). As is the case with wild-type (WT) mice, the *iv/iv* hippocampus contains both ‘ε2-dominant’ and ‘ε2-nondominant’ synapses, and develops apical-basal asymmetry of individual neurons normally. However, the *iv* mouse hippocampus lacks L−R asymmetry; it exhibits right isomerism in the synaptic distribution of the ε2 subunit, irrespective of the laterality of visceral organs (Figure 1, *iv/iv*) [17]. These findings prompted us to carry out behavioral studies using *iv/iv mice*.

In this study, we compared spatial learning and memory between heterozygous (*iv/+*) mice and homozygous *iv*/*iv* mice in a dry maze and a delayed nonmatching-to-position (DNMTP) task. In *iv/+* mice, nodal cilia rotate as rapidly as in WT mice [15], and the asymmetry of both visceral organs and hippocampal circuitry develop normally (Figure 1, *iv/+*) [17]. In the dry maze, mice are trained to search for a food-baited hole in an open field using cues located outside the apparatus. Later, in a single probe trial without baiting food, mice are tested for their ability to locate the food-baited hole. The dry maze is thus essentially the same as the Morris water maze [18], [19], but is considered to be more reliable when comparing different strains and genetic background of mice [20]. The performance during training could be used as a measure for spatial learning, whereas spatial reference memory could be examined by observing the exploratory behavior in the probe trial. On the other hand, the DNMTP is a task in which a mouse is trained to press lever, sustain the memory of the lever location for a short period, and then press the lever opposite to the one the animal pressed earlier in the trial. Accordingly, accuracy in this task thus could be used as a measure of spatial working memory [21]–[23]. The combination of the two tasks enabled us to examine the processes of spatial learning, reference memory and working memory. Here, we report that the *iv/iv* mouse shows deficits in acquisition of spatial navigation and retention of working memory. Our results provide the first direct evidence that L−R asymmetry in hippocampal circuitry is critical for some aspects of higher-order brain functions.

## Methods

### Subjects

A total of 22 male mice were tested in the dry maze: 12 were *iv* (*iv/iv*) mice (SI/Col×C57BL/6 hybrid) and 10 were mice heterozygous for *iv* (*iv/+*) that were produced by crossing *iv/iv* and C57BL/6 mice. Five mice in each group were subsequently tested in a DNMTP task. One *iv/iv* mouse died during the DNMTP training and it was thus excluded from the subsequent analysis of the DNMTP data. The *iv/iv* mice were generated at the Faculty of Science, Kyushu University. At an age of 4 weeks, they were shipped to Tokyo and maintained in the Animal Laboratory at the Department of Psychology, Keio University. The *iv/+* mice were generated and maintained at the Department of Psychology, Keio University. Mice were housed in groups of five; *iv/iv* and *iv/+* were kept in separate cages (29 cm long×19 cm wide×13 cm high) on a 12∶12 h light/dark schedule. Mice were 8 weeks old when the dry maze experiment was initiated. Training was carried out during the dark phase. Mice were kept at or above 85% of their free-feeding weight, which was maintained by supplementary feeding in addition to the food reinforcers in daily testing sessions. Water was freely available in the housing cages. The experiment reported here was conducted in accordance with the guidelines published by the Japan Society for Animal Psychology, and was approved by the Animal Care and Use Committee of Keio University (No. 08007).

### Dry maze

#### Apparatus

The training apparatus was a circular white polypropylene pool with internal dimensions of 89 cm (diameter) and 60 cm (depth). The pool was situated in a laboratory room (170 cm×170 cm×280 cm), elevated 50 cm above the floor. A circular acrylic plate was situated in the pool, 16.5 cm below the top edge of the pool. The plate had 61 holes (1 cm in diameter and 0.5 cm in depth) 10 cm apart from each other. The room contained a digital camera, computer equipment, and furniture in addition to two posters on the walls. These objects served as extra-apparatus cues; the arrangement of these cues remained unchanged throughout the period of experiment. A digital camera (Lifecam VX-7000; Microsoft, Redmond, WA), connected to a computer (Inspiron; Dell, Round Rock, TX) running video tracking software (ANY-maze; Stoelting Company, Wood Dale, IL), allowed observers to watch and record mice with minimal disturbance.

#### Procedure

The basic protocol for the dry maze experiment has been previously described [20]. Each subject first received two sessions of three habituation trials. In the habituation trials, all holes were baited with a 25-mg food pellet (Obara Medical, Tokyo, Japan). Each trial was initiated by placing the mouse on the edge of an open field at one of six starting locations (northwest, west, southwest, south, southeast, and northeast); the trial lasted for 120 s or until the mouse reached one of the baited holes. Subjects that did not locate baited holes were guided to one of the nearest holes and fed with a pellet. During the habituation, each subject experienced all six starting locations. Following the habituation, each subject underwent three sessions of five study trials. In the study trials, only the hole in the northeast location was baited with a pellet. Each trial was initiated by placing the mouse at one of five starting locations (start positions used in the habituation, excluding northeast) randomly chosen without replacement on a trial-by-trial basis. The trial lasted for 120 s or until the mouse located the baited hole. Subjects that did not find the baited hole were guided to it and fed with a pellet, and given a latency score of 120 s. At the end of each trial, each mouse was allowed up to 15 s to consume a pellet on the open field. After each trial, the open field was wiped with 70% ethanol. The intertrial interval (ITI) was around 20–25 min. The mouse received a single probe trial 20–25 min after the final training trial on the last day. In the probe trial, no food was baited and the exploratory behavior of each mouse was recorded for 60 s.

### Delayed nonmatching-to-position task

#### Apparatus

**Figure 2:**
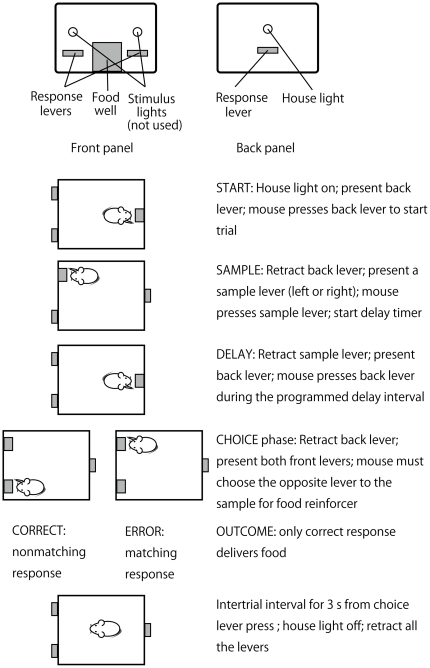
Schematic illustration of apparatus and experimental procedure. (A) The front and back panel of the operant conditioning chamber, and (B) the delayed nonmatching-to-position task sequence.

An operant-conditioning chamber (ENV-307A; Med Associates, Georgia, VT), 21.6 cm long×17.8 cm wide×12.7 cm high (internal dimensions) was used for this task. The chamber was housed in a sound-attenuating box in a test room, and equipped with three retractable levers (ENV-312M): two on the front wall and one on the back (Figure 2A). A 1.0-A house light was positioned above the back lever; two 1.0-A lights, which were not used in the experiment, were positioned above the front levers. A food well was positioned in the center of the front panel; a 25-mg food pellet was delivered into the well by a dispenser (ENV-203-20) to reinforce correct responses. Masking noise was provided by 75-db white noise, which persisted throughout experimental sessions. A Pentium IV computer (Optiplex; Dell, Round Rock, TX) situated outside the testing room controlled and recorded all experimental events and responses via an interface.

#### Procedure

Mice were first trained to perform a sequence of four lever presses when levers were extended into the chamber [23]. The task sequence in DNMTP is illustrated in Figure 2B. Each trial began with the back lever extended. The lever retracted when it was pressed, and one of the two front levers (randomly selected) was extended as the sample for the trial. When the sample lever was pressed, it was retracted, and the back lever was extended. When the back lever was pressed, it was retracted, and both front levers were extended. A press on the opposite lever to the sample (a correct response) was reinforced. A press on the other lever (an incorrect response) ended the trial without reinforcement. When the subject made an incorrect response, the same trial was repeated a maximum of two trials in a row (correction trials). The ITI was 3 s, during which the house light was turned off. Otherwise, the house light was lit throughout the experimental sessions. Each daily session consisted of 40 trials, except for correction trials. When mice did not complete 40 trials after 45 min, the session was terminated. Training was conducted six days a week until 30 sessions had been completed.

After 30 sessions of DNMTP training, the duration of the back lever presentation following the completion of sample lever responses was also gradually lengthened by imposing a set of delays of 1, 3, 5, 7, or 9 s (sessions 31–60), 1, 5, 7, 9, or 13 s (sessions 61–80) and finally extended to 1, 5, 10, 15, 20 s (sessions 81–100). The delays were randomly intermixed without replacement on a trial-by-trial basis. During the delays, back lever-pressing was imposed to prevent mice from using their own positional cues to choose the correct lever. The first press after the elapse of the given delay resulted in retraction of the back lever and extension of both front levers for the choice response. Each daily session consisted of 40 trials. Only the last 20 sessions were used to examine the capacity of working memory.

## Results

### Dry maze

**Figure 3:**
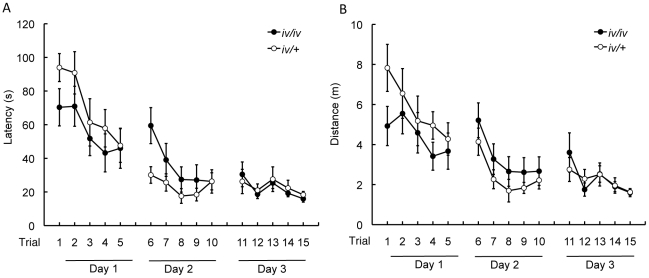
Acquisition of the dry maze. (A) Latency to reach the food-baited hole. Here and elsewhere in the legends, error bar shows the standard error of the mean. (B) Distance traveled to reach the food-baited hole.

During the training trials, both *iv/iv* and *iv/+* mice showed shorter search latency (time to find the baited hole) as the trials proceeded, but *iv/iv* mice improved performance slower than *iv/+* mice (Figure 3A). To examine differences in latency between the groups, we carried out a three-way ANOVA with Group as a between-subject factor and Day and Trial as within-subject factors. During the study trials, the latency became shorter as trials proceeded, within a day as well as across days, in both *iv/iv* and *iv/+* mice, resulting in a significant main effect of Day (*F*_2,40_ = 41.62, *p*<0.001) and Trial (*F*_4,80_ = 9.99, *p*<0.001). The performance approached the asymptotic level toward Day 3, resulting in a significant Day×Trial interaction, *F*_8,160_ = 2.37, *p*<0.05. Although the latency appeared to be shorter in *iv/iv* than *iv/+* mice on Day 1, this tendency was reversed on Day 2, resulted in a significant Group×Day interaction, *F*_2,40_ = 3.73, *p*<0.05. A simple effect for Group×Day interaction was significant only on Day 2, *F*_1,20_ = 4.88, *p*<0.05. Post hoc analysis, using a sequentially rejective test procedure based on Bonferroni inequality, confirmed that *iv/iv* mice continued to improved their performance up to Day 3 (Day 1 vs. Day 2: *t*_11_ = 2.63, *p*<0.05; Day 1 vs. Day 3: *t*_11_ = 4.49, *p*<0.001; Day 2 vs. Day 3, *t*_11_ = 2.75, *p*<0.05), whereas *iv/+* mice reached to the asymptotic level on Day 2 (Day 1 vs. Day 2: *t*_9_ = 5.38, *p*<0.001; Day 1 vs Day 3: *t*_9_ = 8.39, *p*<0.001; Day 2 vs. Day 3: *t*_9_ = 0.15, N.S.).

Performance was also evaluated by measuring traveling distance (Figure 3B). Again, we carried out a three-way ANOVA (Group×Day×Trial) to examine the difference between groups. Overall, the traveling distance was consistent with the latency measures. Both *iv/iv* and *iv/+* mice traveled shorter distances to the baited hole as trials proceeded, within a day as well as across days, resulting in a significant main effect of Day, *F*_2,40_ = 31.30, *p*<0.001, and Trial, *F*_4,80_ = 8.24, *p*<0.001. The performance approached the asymptotic level toward Day 3, resulting in a significant Day×Trial interaction, *F*_8,160_ = 4.26, *p*<0.05. In contrast to the situation regarding latency, a simple effect of Group on Day was not significant, *F*s<3.56. When the traveling distance was examined separately between the two groups, *iv/iv* mice continued to improve performance up to Day 3 (Day 1 vs. Day 2: *t*_11_ = 1.78, N.S.; Day 1 vs. Day 3: *t*_11_ = 3.20, *p*<0.05; Day 2 vs. Day 3: *t*_11_ = 2.12, N.S.), whereas *iv/+* mice reached the asymptotic level on Day 2 (Day 1 vs. Day 2: *t*_9_ = 6.75, *p*<0.001; Day 1 vs. Day 3: *t*_9_ = 8.50, *p*<0.001; Day 2 vs. Day 3: *t*_9_ = 0.78, N.S.).

**Figure 4:**
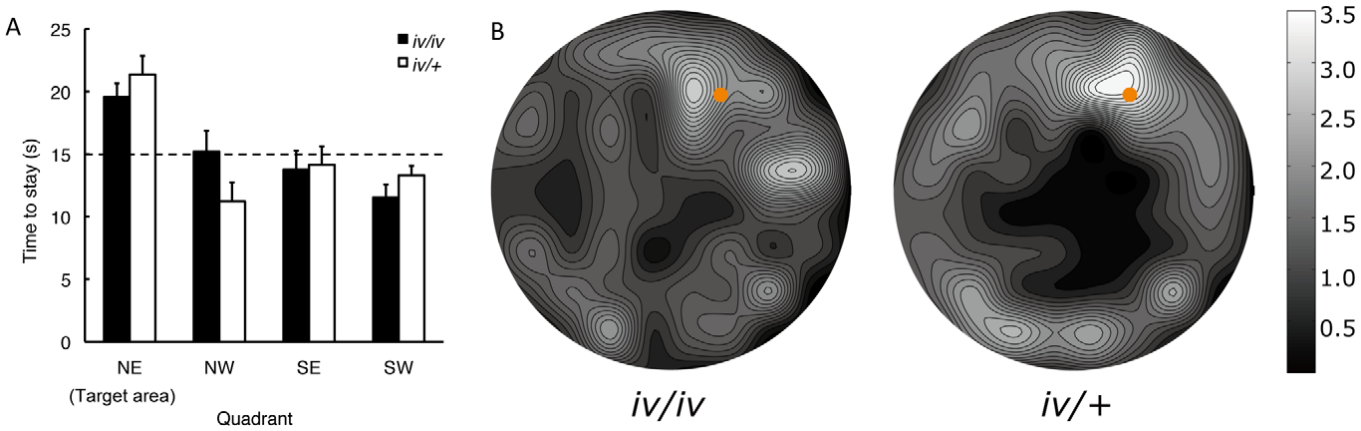
Exploratory behavior during the probe trial of the dry maze. (A) Time spent exploring in quadrants of the open field during the probe trial (NE: north east, NW: north west; SE: south east, SW: south west). NE was the target quadrant, which contained the food-baited hole. A dashed line indicates the prediction from chance. Both groups explored the target quadrant containing the food-baited hole longer than would be expected from chance. (B) The coarse spatial distribution of exploratory behaviors during the probe trial. The open field was fitted into a 10×10 square matrix, and the duration spent in each cell was averaged across subjects and then contour plotted using spline interpolation. Orange circles indicate the location of the food-baited hole during training. The exploratory behavior of the *iv/+* mice was more focused around the baited hole, whereas it was less focused in *iv/iv* mice.

Figure 4A shows the exploring time spent in each quadrant in the open field during the probe trial. Both groups explored the target quadrant containing the food-baited hole longer than would be expected from chance (i.e., 15 s) (one sample t-test, iv/iv: *t*_11_ = 18.14, *p*<0.001; iv/+: *t*_9_ = 14.31, *p*<0.001). The exploring time spent in the target quadrant was, however, not statistically different between the two groups (*t*_9_ = 1.00, N.S.). Figure 4B shows the coarse spatial distribution of exploratory behaviors during the probe trial. The open field was fitted into a 10×10 square matrix, and the duration spent in each cell was averaged across subjects and then contour plotted using spline interpolation. Although no statistical difference was found in the exploration time spent in the target quadrant (Figure 4A), the exploratory behavior of the *iv/+* mice was focused more around the baited hole, whereas it was less focused in *iv/iv* mice (Figure 4B).

### Delayed nonmatching-to-position task

**Figure 5:**
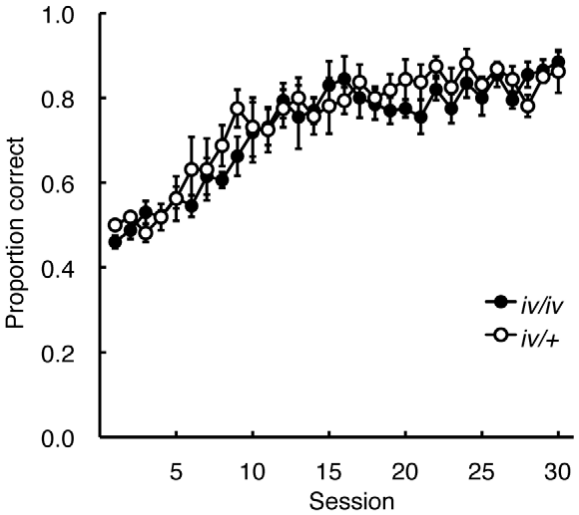
Acquisition of the delayed nonmatching-to-position (DNMTP) in *iv/iv* and iv/+ mice. During DNTP training with a 0-s programmed delay interval, both *iv/iv* and *iv/+* mice acquired the task equally fast.

During DNMTP training with a 0-s programmed delay interval, both *iv/iv* and *iv/+* mice acquired the task equally fast (Figure 5). To examine the difference between the groups, 30 sessions were converted to six 5-session blocks. We then carried out a two-way ANOVA (Group×Session block). A main effect of Session block was significant, *F*_5,35_ = 60.23, *p*<0.001, but a main effect of Group, F_1,7_ = 0.390, and an interaction of the two, *F*_5,35_ = 1,18, were not significant.

**Figure 6:**
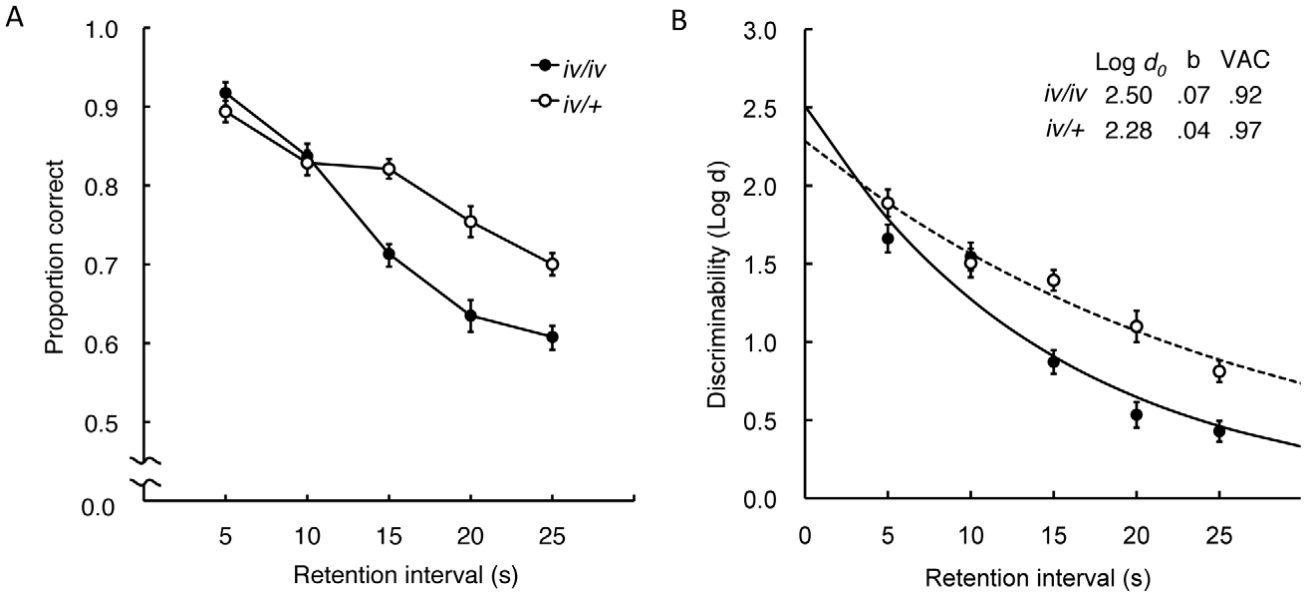
Retention accuracy of the delayed nonmatching-to-position (DNMTP). (A) The delayed nonmatching performance was evaluated using the proportion correct, calculated for 5-s bins of retention intervals (the length of time between sample and comparison responses) for *iv/iv* and *iv/+* mice in the last 20 sessions. (B) Mean discriminability as a function of retention interval for *iv/iv* and *iv/+* mice. Parameter values for the best-fitting exponentials (solid and dashed curves) are given for the two groups. The data are based on the performances in Test 3. Log *d*_0_ indicates the discriminability at 0-s delay and *b* indicates the rate of forgetting. Measures of variance in the data accounted for (VAC) by the best fitting functions by Equation 2 indicate the fitting was satisfactory.

When retention intervals were extended, the proportion correct systematically decreased according to the increase of retention interval in both groups, but more severely in *iv/iv* mice (Figure 6A). A two-way ANOVA (Group×Retention interval) revealed a significant main effect of retention interval, *F*_1,4_ = 99.60, *p*<0.001. *Iv/iv* mice exhibited a lower proportion correct, especially when the retention interval became relatively longer, resulting in a significant main effect of group, *F*_1,38_ = 17.84, *p*<0.001, as well as a significant interaction between group and retention interval, *F*_1,4_ = 11.069, *p*<0.001. Analysis of simple effects for the Group×Retention interval interaction further revealed that *iv/iv* mice performed more poorly than *iv/+* mice at retention interval of 15 s or longer (15 s: *F*_1,38_ = 27.76, *p*<0.001; 20 s: *F*_1,38_ = 17.54, *p*<0.001; 25 s: *F*_1,38_ = 18.27, *p*<0.001).

Delayed nonmatching performance was further analyzed by log *d*, which is a bias-free index of discriminability derived from ratios of correct responses to incorrect responses following different sample stimuli [24]. *d* is calculated using the following formula:
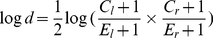
(1)where C and E are the numbers of correct and incorrect responses to left (l) and right (r) comparison levers, respectively. The addition of the constant 1 to the numerator and the denominator allows ratio calculations for cases with no errors. We then calculated the rate of forgetting using the following formula:
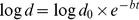
(2)where log *d*_0_ is the discriminability at zero retention interval and *b* is the decay constant of the discriminability as a function of retention interval (t) [25]. The curves shown in Figure 6B were fitted using R, applying least-square nonlinear regression. Little difference was found in log *d*_0_ between the two groups, *t*_38_ = 0.194, N.S., but the forgetting rate constant, *b*, was 1.75 times greater in *iv/iv* than *iv/+* mice, *t*_38_ = 4.91, *p*<0.001.

## Discussion

To investigate the effects of brain laterality defects on spatial learning and memory, we examined the *iv/iv* mouse, which lacks L−R asymmetry of the hippocampal circuitry, using the dry maze and the DNMTP task. The hippocampus is known to be important for the formation of spatial learning and memory in a number of vertebrates, including mouse [18], [19], [26]–[30], and both the dry maze and the DNMTP task are widely used to explore the roles of the hippocampus in spatial learning and memory [31]–[33]. Therefore, we considered that results obtained using *iv/iv* mice reflect a consequence of hippocampal abnormality.

In comparison with *iv/+* mice, *iv/iv* mice improved dry maze performance more slowly, but little difference was found after the performance reached asymptotic level (Figure 3). Patterns of exploratory behavior in the probe trial showed little difference between the two groups when examined by a conventional quadrant distribution analysis (Figure 4A). However, spatial distribution analysis using contour plots (Figure 4B) revealed that *iv/iv* mice did not concentrate their search as close to the target hole location as the *iv/+* mice. These results imply that acquisition of the dry maze is impaired, and that reference memory acquired is less precise, in *iv/iv* mice.

In the DNMTP task, proportion correct was systematically impaired in both *iv/iv* and *iv/+* mice as retention interval increased (Figure 6A). However, the discriminability, estimated by log *d*, was significantly lower in *iv/iv* than *iv/+* mice when retention interval was longer than 15 s. In addition, analysis using negative exponential fitting indicated that the inferior performance of *iv/iv* to *iv/+* mice was due to a relatively large rate constant of the forgetting function (*b*) (Figure 6B). Little difference was found in terms of the initial discriminability (log *d*_0_) between the two groups. This is consistent with our observation that the *iv/iv* mice acquired the delayed nonmatching performance as fast as *iv/+* mice when a 0-s programmed delay interval was used (Figure 5). These results suggest that working memory decays faster in *iv/iv* than *iv/+* mice, but a similar level of L−R discriminability could be acquired by the two groups when no memory load was imposed.

The group differences observed in the present experiments were not caused by physical defects in *iv/iv* mice, but rather by deficits in their spatial learning and memory functions. Dry maze performance at the asymptote did not differ between the two groups (Figures 3A and 3B), implying that the locomotor ability in *iv/iv* mice was not impaired. Moreover, *iv/iv* mice acquired the DNMTP task as fast as *iv/+* mice (Figure 5), further implying that the behavior of *iv/iv* in the operant-conditioning chamber was not impaired.

In conclusion, the asymmetry defect of the *iv* mice hippocampus impairs the acquisition of reference memory and the retention of working memory. Using *in vitro* experiments, our previous studies have demonstrated the Σ2 asymmetry of the hippocampal circuitry as well as the asymmetry defect of the *iv/iv* mouse hippocampus [5], [6], [17]. This study provides the first evidence for a link between hippocampal asymmetry and higher-order brain functions. These findings indicate that the hippocampal asymmetry is a useful model system for exploring the molecular basis of brain asymmetry.
